# Contributing Factors and Evolution of Impulse Control Disorder in the Luxembourg Parkinson Cohort

**DOI:** 10.3389/fneur.2020.578924

**Published:** 2020-11-12

**Authors:** Sylvia Binck, Claire Pauly, Michel Vaillant, Geraldine Hipp, Manon Gantenbein, Rejko Krueger, Nico J Diederich

**Affiliations:** ^1^Luxembourg Centre for System Biomedicine, University of Luxembourg, Luxembourg, Luxembourg; ^2^Centre Hospitalier de Luxembourg, Luxembourg, Luxembourg; ^3^Luxembourg Institute of Health, Luxembourg, Luxembourg

**Keywords:** impulse control disorder, dopamine agonists, Parkinson's disease (PD), risk factors, longitudinal analysis

## Abstract

**Background:** To establish the frequency of impulse control disorder (ICD) in Parkinson's disease (PD).

**Methods:** Within the Luxembourg Parkinson's Study, PD patients were evaluated for ICD presence (score ≥ 1 on MDS-UPDRS I item 1.6), use of dopamine agonists (DA) and other medications.

**Results:** 470 patients were enrolled. Among 217 patients without DA use, 6.9% scored positive for ICD, vs. 15.4% among 253 patients with DA use (*p* = 0.005). The regression analysis showed that age at PD diagnosis had only a minor impact on ICD occurrence, while there was no influence by gender or co-medications. The longitudinal study over 2 years in 156 patients demonstrated increasing ICD frequency in DA users (*p* = 0.005).

**Conclusion:** This large and non-interventional study confirms that PD patients with DA treatment show higher frequency of ICD than patients without DA use. It newly demonstrates that ICD can develop independently from age, gender, or co-medications.

## Introduction

Impulse control disorder (ICD) has been described by the World Health Organization (WHO) in 1992 as “repeated acts that have no clear rational motivation, generally harm the person's own interests and those of other people, and are associated with impulses the person experiences as uncontrollable” (1).

Following the WHO definition, four types of ICD have been defined: binge eating, excessive shopping, pathological gambling and hypersexuality (2, 3). Pilot studies on ICD in PD have also proposed to classify hobbyism and sometimes punding under the ICD umbrella (4, 5). Although the latter behaviors may also reflect perturbed impulse-control, they are less strictly defined and are frequently overlapping with normal behavior (6).

The dopamine dysregulation syndrome characterized by excessive and addictive use of dopaminergic medication can be more easily dissected, as in contrast to ICD, it overwhelmingly is due to excessive use of levodopa and not dopamine agonists (6, 7).

The frequency of ICD in *de novo* PD patients is similar to the general population (5), but numerous studies reported higher frequency during the course of the disease (8–12). Treatment by dopamine agonists (DA), mainly through stimulation of the D3- receptor (13) has been proposed as a causal factor.

However, there is ongoing debate on other risk factors, as not every PD patient treated by DA develops ICD. Previous studies essentially recruited younger PD patients or those with a more rapid disease progression in tertiary care centers (5, 9, 11). Therefore, these studies cannot extrapolate their findings to the general PD population. Potential further risk factors such as age at PD diagnosis, gender, genetic susceptibility factors, comorbidities, or co-medication have been poorly addressed (14). Whether the dopamine agonist specifically or the dopamine replacement therapy in general, reflected by the levodopa equivalent daily dosis (LEDD), is implicated in the development of ICD, is also subject of discussion. Finally, the development of ICD in PD over time has only been explored in a few longitudinal studies (5, 9, 11).

The present study could successfully circumvent some of these reservations. The Luxembourg Parkinson's study (15) was designed to be as inclusive as possible by active recruitment of also elderly participants or those with reduced mobility, thus best representative of the general PD population (16). This allows our study to produce statistically robust data on most of these so far debatable issues.

## Methods

### Cohorts of PD Patients and Controls

PD patients were recruited from the Luxembourg Parkinson's study (15). In this descriptive, longitudinal, prospective study, participants benefit from detailed annual follow-up examinations.” Subjects were eligible for the study when fulfilling the following criteria: PD diagnosis based on the UK PD Society Brain Bank criteria (17) and a score of ≥18 points on the Montreal Cognitive Assessment (MoCA) (18) in order to ensure sufficient comprehension of the questionnaires used. Patients with atypical PD were excluded. Based on DA treatment, patients were divided in two groups: “DA-users” (DA+) and “non DA-users” (DA–). They were adjusted for age and gender. Controls were also recruited from the Luxembourg Parkinson's study (15). They only had a baseline assessment and were adjusted for age at study entry with patients classified as DA–. We excluded controls with any suspicion of incident PD or under DA treatment for other medical reasons. For the longitudinal analysis in PD patients, we only considered those who did not secondarily convert into atypical Parkinsonism.

### Assessments

For each enrolled subject we assessed the demographic data and the current medication, including levodopa, DA, amantadine and antidepressants. Based on the indicated dosages of all dopaminergic agents, a levodopa equivalent daily dose was calculated (LEDD) (19). Previous dosages of the present medication were not assessed. We evaluated the MDS-UPDRS part I-IV (20) and the modified Hoehn and Yahr scale (21).

Similarly to another study on ICD occurrence in PD (5), ICD was evaluated by assessing MDS-UPDRS part I item 1.6. This item quantifies ICD with a score ranging from zero (normal) to four (severe). We defined the presence of ICD by a score ≥ 1, as previously proposed (5).

Of note, the proposed text of the MDS-UPDRS part I item 1.6 was explained in detail to the patient, with the examiner insisting on the five above-mentioned symptoms of ICD. Any allusion to punding as well as excessive and addictive intake of the dopaminergic medication were disregarded.

### Statistical Analyses

The study data were managed by using the Redcap electronic data capture tools (22). The statistical analyses were performed using SPSS Statistics version 25 and SAS V9.3 (SAS Institute, Cary, NC, USA). All tests were two-sided and *p* ≤ 0.05 were considered statistically significant. Assumption of normality was tested by the Shapiro-Wilk test. ICD frequency was calculated by a chi square test and demographic variables also by the Mann-Whitney U-test as adequate. We performed a logistic regression model to estimate the relationship between ICD presence and confounding factors by putting them all into one model. In a second analysis, interactions between DA use and the annual visits V1 and V3 were tested. In both models, adjustments on gender and age were implemented.

All patients had given written consent before entering the Luxembourg Parkinson's study (15). The study was approved by the National Ethics Board (CNER Ref: 201407/13) and the National Data Protection Committee (CNPD Ref: 446/2017).

## Results

### Baseline Characteristics

Between March 2015 and December 2019, 470 patients (mean age 66.4 [39.4–86.4] years) and 429 controls (mean age 66.8 [55.2–89.5] years) were eligible for the study. Two hundred and fifty three patients (54%) were classified DA+ and 217 DA– (mean age 66.6 [51–86] vs. 66.9 years [39–80], *p* = 0.2). DA+ were younger at PD diagnosis than DA– (58.5 [27.2–78.4] vs. 61.2 [31.3–79.4] years; *p* = 0.001) and had a longer disease duration (8.1 [0–34] vs. 5.7 [0–26] years, *p* < 0.001). The complete comparison of the demographic data is shown in Table 1.

**Table 1:** Demographics and medication profile in 470 patients (253 DA+, 217 DA–) and 429 controls.

	**All PD patients**	**PD DA+**	**PD DA-**	***p*-value DA+ vs. DA–**	**Controls**	***p*-value PD DA– vs. controls**
Subjects	470	253	217		429	
**Demographics**						
Male sex (%)	308 (65.5%)	156 (61.7%)	152 (70.0%)	0.06	234 (54.5%)	<0.001^**^
Years of education (yrs.)	13.0 (±3.9)	13.1 (±3.6)	12.9 (±4.3)	0.70	13.8 (3.7)	0.01^*^
MoCA	25.2 (±3.1)	25.4 (±3.0)	24.9 (±3.2)	0.11	26.9 (2.5)	<0.001^**^
Age at baseline (yrs.)	66.8 (±8.5)	66.6 (±8.0)	66.9 (±9.0)	0.18	66.8 (8.5)	0.35
Age at PD onset (yrs.)	59.8 (±9.9)	58.5 (±9.3)	61.2 (±10.4)	0.001^**^	n.a	n.a
PD duration (yrs.)	6.9 (±5.5)	8.0 (±5.5)	5.7 (±5.2)	<0.001^**^	n.a	n.a
LEDD (mg)	559 (±441)	696 (±429)	397 (±399)	<0.001^**^	0	n.a
**Scores**						
MDS-UPDRS I 1.6 = 0 (*n*)	416 (88.5%)	214 (84.6%)	202 (93.1%)	n.a	409 (95.4%)	n.a
MDS-UPDRS I 1.6 ≥ 1 (*n*)	54 (11.5%)	39 (15.4%)	15 (6.9%)	n.a	20 (4.6%)	n.a
MDS-UPDRS III (score)	33.2 (±14.4)	33.2 (±15.3)	33.2 (±13.3)	0.8	3.7 (4.0)	n.a.
Hoehn & Yahr (score)	2.1 (±0.9)	2.2 (±0.7)	2.1 (±0.6)	0.61	0	n.a
**Medication**						
Amantadine (*n*)	81 (17.2%)	48 (19.0%)	33 (15.2%)	n.a	n.a	n.a
Antidepressants (*n*)	64 (13.9%)	34 (13.2%)	30 (14.4%)	n.a	20 (4.6%)	n.a
Ropinirole (*n*)	63 (13.4%)	63 (26.8%)	0	n.a	0	n.a
Pramipexole (*n*)	146 (31.1%)	146 (62.2%)	0	n.a	0	n.a
Rotigotine (*n*)	32 (6.8%)	32 (13.6%)	0	n.a	0	n.a
Apomorphine (*n*)	1 (0.6%)	1 (1.2%)	0	n.a	0	n.a
Piribedil (n)	16 (3.4%)	16 (6.8%)	0	n.a	0	n.a

p-values concern differences between DA+ and DA– (

* *p < 0.05*,

** *p < 0.01)*.

*Some patients take >1 DA, which explains >100% in the DA+*.

### ICD Frequencies

Based on the answers to the assessment, ICD frequency was higher in PD patients than in controls (11.5 % vs. 4.6%; *p* < 0.001). This difference was probably due to a higher positive scoring in DA+ (39 out of 253 DA+ [15.4%] vs. 15 out of 217 DA- [6.9%], *p* = 0.005).

Fifty four patients in total scored positive for ICD (38 males and 16 females). The most common ICD subtype in men was binge eating and in women excessive shopping. The detailed data about ICD subtypes and male-female distribution can be found in Table 2.

**Table 2:** ICD subtypes with male-female distribution in subjects with MDS-UPDRS ≥ 1 at baseline (54 patients).

	**Male (*n* = 30)**	**Female (*n* = 10)**	***p*-value**
Binge eating (*n*)	12	2	*p* = 0.14
Excessive shopping (*n*)	7	4	*p* = 0.58
Pathological gambling (*n*)	0	2	*p* = 0.03^*^
Hypersexuality (*n*)	6	0	*p* = 0.09
Hobbying (*n*)	5	2	*p* = 0.95
Total (*n*)	30	10	

* *p ≤ 0.05 is considered statistically significant*.

Twenty one Patients with advanced treatment (19 with Deep brain stimulation, two with apomorphine or Duodopa-pump) were included. Three patients with DBS scored positive for ICD. There was no statistically significant association between DBS treatment and ICD symptoms (*p* = 0.55).

Notably, there was no significant difference in ICD frequency between DA- patients and controls (6.9% vs. 4.6%, *p* = 0.21).

### Potential ICD Risk Factors

We analyzed the potential influence of ICD risk factors by a logistic regression model (Supplementary Table 1). We found that younger age at disease onset independently affected ICD occurrence (*p* = 0.03). We also found that the higher the age of onset, the higher the ICD frequency. The odds ratio was borderline significant (OR = 1.003, 95%CI= [1.000; 1.006]). There was no influence of gender (*p* = 0.1), disease duration (*p* = 0.2), UPDRS III (*p* = 0.06), use of amantadine (*p* = 0.5) or antidepressants (*p* = 0.5). Despite the fact that LEDD was higher in DA+ than in DA– (696 [0–1625] vs. 397 mg [0–2112], *p* < 0.001) our model did not show an association (*p* = 0.2). In a next step, we adjusted both patient subgroups for age at PD diagnosis: 242 DA+ and 203 DA–. The difference in ICD frequency was maintained (14.9% vs. 7.4, *p* = 0.02). In a final step, we separately analyzed ICD occurrence in 285 PD patients older than 65 years at study entry: 145 DA+ and 140 DA– patients. Here, the difference was also maintained (12.5% vs. 4.3%, *p* = 0.01).

### Longitudinal Follow-Up of ICD

**Figure 1:**
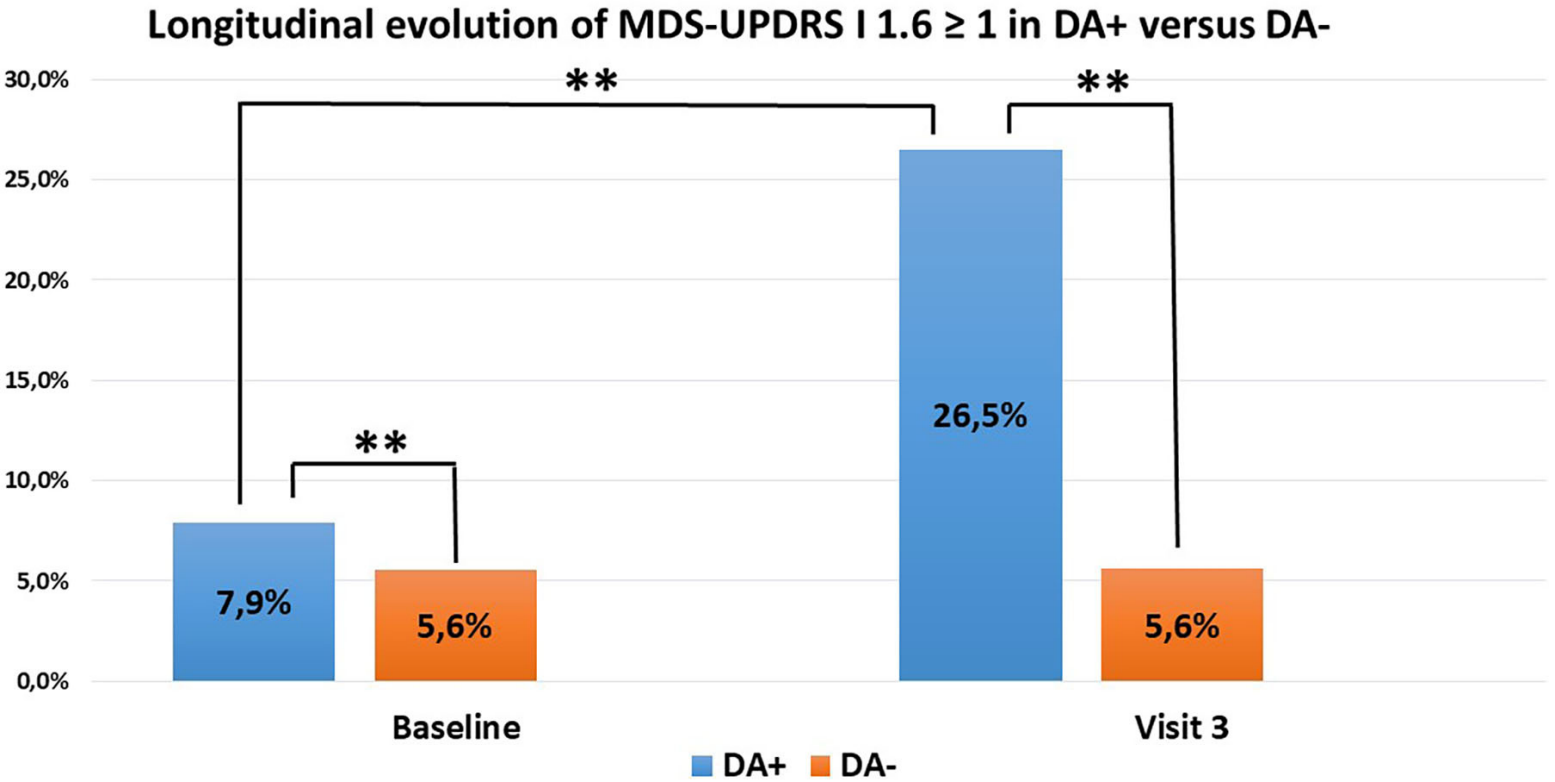
ICD evolution in PD patients (102 DA+, 54 DA–) with unchanged DA group adherence over time. DA+ in blue, DA– in orange. Increase of ICD in DA+ between baseline and V3. Difference between DA+ and DA– (**p < 0.01).

The database identified 193 patients having accomplished visits V1 to V3. Three patients were excluded because of conversion into atypical parkinsonism during follow-up (one patient was reclassified as Lewy Body Dementia and two as secondary parkinsonism). We based our analysis on the 156 patients with unchanged DA group adherence from baseline to V3: 102 PD patients in the DA+ subgroup and 54 patients in the DA- subgroup. There was a sharp increase from baseline to V3 in the DA+ subgroup (7.9% vs. 26.5%, *p* < 0.001). In contrast, ICD frequency remained stable in the DA- group (5.6%). When comparing ICD occurrence between both groups at V3, the difference was again significant (26.5% vs. 5.6%, *p* < 0.001) (Figure 1). Of note, we did have participants with a longer follow-up period (up to 4 years). However, statistical robustness of a longitudinal model was not achieved because of insufficient subjects and these data are not shown.

## Discussion

This study confirms a higher ICD frequency in DA+ than in DA- PD patients. As frequency in DA- is similar to the frequency in age-adjusted controls, ICD is not a PD-inherent syndrome. Age at PD onset is a significant co-factor, but age at study entry is not. Remarkably, ICD frequency remains significantly higher in aged PD patients treated by DA compared to those not treated by DA. Our study argues against the initial concepts that ICD incidence would increase with male gender, or higher motor deficits (9, 23).

It has been proposed that LEDD plays a role as a risk factor, which is not supported by our data. In our cohort, the total LEDD contains a substantially higher part of levodopa than of DA. However, in contrast to DA, levodopa does not preferentially stimulate the D3– receptor imputed in generation of ICD symptoms by its impact on cognitive and emotional functions (13). In addition, amantadine, prescribed in 17% of the patients in this cohort, did not show any protective effect against ICD occurrence (24, 25).

There has also been interest in a potential genetic predisposition for ICD in PD. In one study, the dopamine D3 receptor Ser9Gly variant has been linked to higher prevalence of ICD (26). Two variants of the opioid receptor OPRM1 (rs17999 and rs702764) have been associated with decreased odds of ICDs (27, 28). Mutations of the PINK1 and Parkin have been identified as risk factors for ICD (29, 30). All these results await confirmation in independent, larger cohorts.

In the longitudinal analysis, we observed a sharp increase of ICD frequency in DA+ between baseline and V3. We have no data on DA treatment duration and DA dosage before study entry, so we cannot explicitly dissect if this increase is a time or a dosage effect, reflecting DA accumulation over the years. The latter seems more probable although there may exist an individually variable “tipping point” in terms of postsynaptic dopaminergic hypersensitization (13).

It is appealing to compare our data with other longitudinal studies from Europe, although most of them had a cross-sectional design and were conducted in tertiary care centers. However, studies with a longitudinal follow-up design remain rare. The DIGPD study (France) (5) and the ICARUS study (Italy) (9) are both large, multicenteric cohorts. The DIGPD study (5) included 411 patients (average follow-up 3.3 years); an increase of ICD from 19.7% at baseline to 32.8% after 5 years was reported. With 1,069 subjects included, the ICARUS study (9) reported an ICD prevalence of 26.8% after a 2 year follow-up.

In the U.S., the Prospective Cohort Study of Impulse Control Disorders in Parkinson's disease (11) longitudinally screened 164 subjects for ICD over 4 years. 39.1% of subjects with DA treatment developed ICD after an average of 21 months. In comparison to these studies, the frequency of ICD at baseline and after 2 years in our study is rather low. This could be due to a more cautious use of DA by the clinicians, based on their acquired knowledge of risky ICD behavior and, in particular, to the more restrictive definition of ICD in our study. However, the lower frequency in the present study probably better reflects numbers expected in the general PD population, but at the same alerts the clinician of increasing frequency of ICD over time.

Several strengths of this study have to be outlined. First, the recruitment of PD patients without any limitations imposed by age or comorbidities allowed a broader appreciation of ICD in the general PD population than in three other studies (5, 9, 11). With a mean age of 65 years, PD patients in our study are also substantially older than those studies (5, 9, 11) (mean age 58, 59 and 62 years, respectively). Second, the inclusive Luxembourg Parkinson's study (15) design also circumvented the preferential recruitment of patients in need of advanced treatment. Third, the physicians in the present observational study were not prescribing nor interfering with treatment. Finally, we were able to follow a large and unselected subgroup of patients longitudinally over three visits, as rarely done before and not at this scale.

Our study has several limitations: first, comparably to other studies (5, 11), ICD occurrence was not confirmed by a second test and defined by MDS-UPDRS part I item 1.6.

In particular, we did not *systematically* use the Questionnaire for Impulsive-Compulsive Disorders in Parkinson's disease (QUIP) (31), as it is subject to false positive findings. Due to antidepressant use (12), it can produce up to 40% false positive results due to an over-identification or an experience of subsyndromal symptoms (32). Second, we have no reliable data on the medication used before study entry. Finally, our dataset did not allow comparing different DA release forms as lower ICD frequencies have been reported with transdermal DA treatment (33).

In conclusion, our study shows that in PD patients there is a strong association between ICD occurrence and DA treatment. It is independent from gender and age at study entry. Our study cannot robustly show if there is a gender impact on the ICD subtype.

ICD frequency substantially increases over time. Preliminary results from visits 4 and 5 similarly show a trend for ascending ICD frequency over time, but the data obtained so far are insufficient for valid statistical interpretation.

Future research has to identify the type of DA application at highest risk for ICD (34). Analyzing cognitive, affective and motivational correlates or initial PD manifestations could be of interest as well (35). Possibly, the most promising path will be the identification of distinct genotypes that increase the risk for ICD (29, 30). Physicians prescribing DA treatment have to be attentive to the risk of these side effects in *any* PD patient, independent from disease duration and age. They should regularly re-interview PD patients on ICD occurrence, as early ICD absence does not preclude later manifestation.

## Data Availability Statement

Requests to access the datasets should be directed to the coordinator of the NCER-PD research programme, email: rejko.krueger@uni.lu.

## Ethics Statement

The studies involving human participants were reviewed and approved by the National Ethics Board (CNER Ref: 201407/13) and the National Data Protection Committee (CNPD Ref: 446/2017). The patients/participants provided their written informed consent to participate in this study.

## Author Contributions

SB and ND: conception of research project. ND: organization of research project. SB, CP, and GH: execution of research project. SB and RK: supervision of research project. MV: design of statistical analysis. CP and MV: execution of statistical analysis. MV and ND: review and critique of statistical analysis. SB, CP, and ND: writing of the first draft of manuscript preparation. CP, MV, GH, RK, and ND: review and critique of manuscript preparation. All authors contributed to the article and approved the submitted version.

## Conflict of Interest

The authors declare that the research was conducted in the absence of any commercial or financial relationships that could be construed as a potential conflict of interest.

## Abbreviations

DA: dopamine agonist
DA+: DA-users
DA-: non DA-users
ICD: impulse control disorder
LEDD: levodopa equivalent daily dosage
MDS-UPDRS: Movement Disorder Society Unified Parkinson's Disease Rating Scale
PD: Parkinson's disease
V1: Visit 1
V3: Visit 3.
