# Corrigendum: Contributing Factors and Evolution of Impulse Control Disorder in the Luxembourg Parkinson Cohort

**DOI:** 10.3389/fneur.2020.638735

**Published:** 2021-01-12

**Authors:** Sylvia Binck, Claire Pauly, Michel Vaillant, Geraldine Hipp, Manon Gantenbein, Rejko Krueger, Nico J Diederich

**Affiliations:** ^1^Luxembourg Centre for System Biomedicine, University of Luxembourg, Luxembourg, Luxembourg; ^2^Centre Hospitalier de Luxembourg, Luxembourg, Luxembourg; ^3^Luxembourg Institute of Health, Luxembourg, Luxembourg

**Keywords:** impulse control disorder, dopamine agonists, Parkinson's disease (PD), risk factors, longitudinal analysis

In the original article, we neglected to include the Acknowledgments section. The Acknowledgments section should appear as shown below.

We acknowledge the joint effort of the National Centre of Excellence in Research on Parkinson's Disease (NCER-PD) consortium members generally contributing to the Luxembourg Parkinson's Study as listed below:

Geeta Acharya, Gloria Aguayo, Myriam Alexandre, Dominic Allen, Wim Ammerlann, Maike Aurich, Federico Baldini, Rudi Balling, Peter Banda, Katy Beaumont, Regina Becker, Camille Bellora, Daniela Berg, Fay Betsou, Sylvia Binck, Alexandre Bisdorff, Dheeraj Bobbili, Kathrin Brockmann, Jessica Calmes, Lorieza Castillo, Nico Diederich, Rene Dondelinger, Daniela Esteves, Jean-Yves Ferrand, Ronan Fleming, Manon Gantenbein, Thomas Gasser, Piotr Gawron, Lars Geffers, Virginie Giarmana, Enrico Glaab, Clarissa Gomes, Nikolai Goncharenko, Jérôme Graas, Mariela Graziano, Valentin Groues, Anne Grünewald, Wei Gu, Gaël Hammot, Anne-Marie Hanff, Linda Hansen, Maxime Hansen, Hulda Haraldsdöttir, Laurent Heirendt, Estelle Henry, Sylvia Herbrink, Johannes Hertel, Sascha Herzinger, Michael Heymann, Karsten Hiller, Geraldine Hipp, Michele Hu, Laetitia Huiart, Alexander Hundt, Nadine Jacoby, Jacek Jarosław, Yohan Jaroz, Pierre Kolber, Rejko Krüger, Joachim Kutzera, Pauline Lambert, Zied Landoulsi, Catherine Larue, Roseline Lentz, Inga Liepelt, Robert Liszka, Laura Longhino, Victoria Lorentz, Paula Cristina Lupu, Clare Mackay, Walter Maetzler, Katrin Marcus, Guilherme Marques, Jan Martens, Piotr Matyjaszczyk, Patrick May, Francoise Meisch, Myriam Menster, Maura Minelli, Michel Mittelbronn, Brit Mollenhauer, Kathleen Mommaerts, Carlos Moreno, Friedrich Mühlschlegel, Romain Nati, Ulf Nehrbass, Sarah Nickels, Beatrice Nicolai, Jean-Paul Nicolay, Alberto Noronha, Wolfgang Oertel, Marek Ostaszewski, Sinthuja Pachchek, Claire Pauly, Lukas Pavelka, Magali Perquin, Dorothea Reiter, Isabel Rosety, Kirsten Rump, Estelle Sandt, Venkata Satagopam, Marc Schlesser, Margaux Schmitt, Sabine Schmitz, Susanne Schmitz, Reinhard Schneider, Jens Schwamborn, Alexandra Schweicher, Kate Sokolowska, Lara Stute, Ines Thiele, Cyrille Thinnes, Christophe Trefois, Jean-Pierre Trezzi, Johanna Trouet, Michel Vaillant, Daniel Vasco, Maharshi Vyas, Richard Wade-Martins, and Paul Wilmes.

The authors apologize for this error and state that this does not change the scientific conclusions of the article in any way. The original article has been updated.

