# Supplementary material for: Contributing Factors and Evolution of Impulse Control Disorder in the Luxembourg Parkinson Cohort
Source: Front Neurol. 2020 Nov 12;11:578924. doi: 10.3389/fneur.2020.578924 (PMC7688665; doi:10.3389/fneur.2020.578924)
Supplement: Supplementary file 1 [file Data_Sheet_1.PDF]

	B	S.E.	Wald	df	Sig.	Exp(B)	Lower CI	Upper CI
Gender	1.582	1.052	2.261	1	.133	4.866	.619	38.266
PD_Duration	-.199	.148	1.824	1	.177	.819	.613	1.094
Levodopa Equivalent Daily Dose (LEDD)	-.084	.063	1.746	1	.186	.920	.812	1.041
Age_at_PD diagnosis	.003	.001	5.083	1	**.024**	1.003	1.000	1.006
Amantadine	-.852	1.227	.483	1	.487	.426	.039	4.719
UPDRS Total Score Part III:	.066	.035	3.607	1	.058	1.068	.998	1.142
Antidepressants	.068	.094	.535	1	.464	1.071	.891	1.286
Constant	-2.555	4.316	.350	1	.554	.078		

Regression model on the influence of the different risk factors on ICD frequency. B = intercept; S.E. = standard error; df = degrees of freedom; Sig. = significance; Exp (B) =exponentiation of the B coefficient; CI = confidence interval (95%).
